# Rise of the Machines

**DOI:** 10.1371/journal.pgen.1000134

**Published:** 2008-08-01

**Authors:** David Gresham, Leonid Kruglyak

**Affiliations:** 1 Lewis-Sigler Institute for Integrative Genomics, Princeton University, Princeton, New Jersey, United States of America; 2 Department of Molecular Biology, Princeton University, Princeton, New Jersey, United States of America; 3 Department of Ecology and Evolutionary Biology, Princeton University, Princeton, New Jersey, United States of America; The Jackson Laboratory, United States of America

Until recently, sequencing the entire genome of an organism was a major endeavor. New technologies are transforming this task into routine practice and launching a new assault on whole-genome sequencing.

It is more than 30 years since Sir Fred Sanger and colleagues published their method for sequencing DNA [1]. This Nobel Prize–winning work formed the basis of the vast majority of subsequent sequencing methodologies, albeit with some crucial technical innovations. Despite the great utility of Sanger sequencing, its scalability is inherently limited, and therefore the creation of warehouse-sized facilities was required to accomplish whole-genome sequencing projects. As a result, sequencing more than a few kilobases of DNA—a requirement for all but the simplest genomes—has long remained the province of a few dedicated sequencing centers. Within the last year, however, things have begun to change in dramatic ways. New sequencing technologies are emerging, announced in an assortment of reports, conference presentations, and press releases. In this issue of *PLoS Genetics*, Srivatsan et al. [2] report the resequencing of several genomes of the bacterium *Bacillus subtilis* using one of these new technologies. A new battle at the frontier of DNA sequencing has commenced.

## Not Alone Anymore

The monopoly enjoyed by Sanger sequencing is coming to an end. New technologies have recently emerged, including the Illumina Genome Analyzer (formerly Solexa sequencing), the Genome Sequencer FLX System (formerly 454 sequencing), and the ABI SOLiD System. Each of these machines uses different and entirely new methods for sequencing DNA. However, their commonality lies in simultaneously capturing millions of sequence stretches (reads) of comparatively short length (25–200 base pairs). Due to the short read length, a reference sequence is usually required to guide the genome assembly. How this approach to resequencing whole genomes works in practice is sensibly vetted in model organisms.

Remarkably, since the original publication of the relatively small (4.2 Mb) *B. subtilis* genome over 10 years ago [3], only one genome sequence of this organism has been available—that of the laboratory strain 168. The paper by Srivatsan et al. [2] increases the number of sequenced *B. subtilis* genomes by an order of magnitude. Using an Illumina Genome Analyzer, the authors resequenced the genome of the same isolate of strain 168 used to generate the original reference genome. Generating over 5 million sequencing reads of 36 bp each, 87% of which could be mapped to the genome, the authors achieved an average of 40-fold coverage. Using recently developed algorithms to align the reads to the reference sequence [4] and to generate de novo genome assemblies [5],[6], the authors identified a surprisingly high number of sequence discrepancies throughout the genome (1,519 base substitution, 82 insertions, and 85 deletions) compared with the original reference (i.e., a total sequence difference of 0.04%). Follow-up analyses indicated that the vast majority of the discrepancies reflected errors in the original reference sequence.

Typically, reference genome sequences represent a single, commonly used lab strain. To explore genomic diversity among different lab strains, Srivatsan et al. resequenced another independent isolate of strain 168 as well as different isolates of three other commonly used lab strains. The results emphasize the fact that in model organisms, different strains are often significantly diverged at the nucleotide level [7]. In the most extreme case, sequencing new strains can reveal completely novel genome features, such as an apparent unique 78-kb plasmid [8], which the authors identified in the sequence data of one *B. subtilis* strain. Sequencing different isolates of the same strain illuminates the fact that individual isolates that are “isogenic” can differ by many nucleotides. Divergence among strains that are genetically isolated for many generations in different laboratories is likely to exist for all model organisms, from bacteria to mice [9].

Whole-genome resequencing has the potential to dramatically reduce the task of connecting genotype to phenotype. Srivatsan et al. provide two such examples: they identified a previously unappreciated deficiency in citrate metabolism in one lab strain, and they uncovered genetic interactions among three genes that mediate the stringent response to starvation. In the latter case, the authors resequenced the genome of a *rel*A knockout strain harboring extragenic suppressors of the *rel*A growth defect. They identified mutations in two genes, with each partially suppressing the *rel*A deletion phenotype, but with full suppression only achieved when both genes are mutated. These results, along with previous work in yeast using genome tiling arrays for comprehensive mutation detection [10], hint at the enormous potential of genome resequencing to revolutionize genetic screens for mutants, suppressors, and enhancers by drastically accelerating the previously rate-limiting step of detecting one or a few mutations in an entire genome. This task was previously limited to certain classes of mutations that were easier to detect, such as transposon insertions and large deletions, or required laborious mapping and cloning, which was possible only in organisms that are good genetic systems. Genome resequencing will make genetic screens feasible for all classes of mutations, for a vastly expanded range of organisms, for phenotypes that are subtle, prohibitive to measure in many individuals, or unstable due to rapid acquisition of suppressors, and under many other previously intractable scenarios.

A burning question is how the short-read approach to genome resequencing can be scaled to larger and more complex genomes. A number of technical questions remain that are not addressed by this study, such as how heterozygosity confounds the analysis, whether there are systematic errors and biases in the data, and how to surmount the problem of reads falling within repetitive DNA sequence. A previous study in *Caenorhabditis elegans* [11] excluded any repetitive sequence larger than the Illumina Genome Analyzer read length, which meant that ∼25% of the genome was not examined. Although Srivatsan et al. appear to have met reasonable success with de novo assembly, it is not clear that this would work in the same way with a more complex genome.

## An Altered Landscape

**Figure 1:**
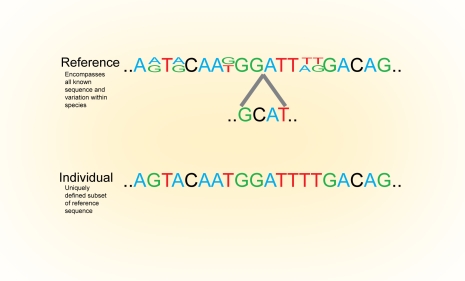
Conceptualizing the Genome. The determination of many thousands of genomes will require a precise definition of what a genome sequence represents. We envisage a hierarchy in which a reference genome comprising all known sequences within a species is placed at the topmost level. A subset of the reference sequence defines a strain or population genome that includes all known polymorphisms within the population. At the individual level is a uniquely defined genome. Such a defined hierarchy would facilitate unique identifiers for classes within each level (for example, for a microbial isolate a Unique Genome Identifier could take the form XXX-YYY-ZZZ, where XXX denotes the species, YYY denotes the strain, and ZZZ denotes the specific isolate sequenced). This hierarchy would also enable efficient data storage of complete genome information for individuals, because the information stored at a lower level of specification needs only to describe what is specific to that level.

Enabling technologies often present us with new issues, some of which are foreshadowed by the current report [2]. Resequencing new individuals raises the question of how we should define a reference genome (see Figure 1). Today, reference genomes—including the human genome [12]—derive from either one individual (or strain) or a composite of sequences from a small number of individuals. As more individuals are sequenced and differences are revealed, the reference genome should not cling to its historical underpinnings but rather should reflect the acquisition of new knowledge. Errors in the original reference should obviously be corrected, but true genetic diversity must not be swept under the rug. At its most extreme, new genes will be identified that are completely absent in the original reference. Clearly, these should be added to an organism's reference genome. The reference genome must evolve from its current form to a “meta-genome,” which includes the superset of all sequences identified in an organism.

As entire genome sequences are determined from multiple individuals (from the same species and population or strain background), we will require new language and tools to categorize, annotate, and archive these different genomes and to clearly describe their relationship to the reference “meta-sequence.” The publication of these genomes will require detailed accompanying information on the provenance of the sequenced DNA, and it will become increasingly important to adhere to guidelines for reporting whole-genome information, such as those recently proposed [13].

It is clear that whole-genome resequencing will be of immense value in connecting genotype to phenotype. However, resequencing should not be considered a panacea for biological questions. Experimental designs that aim to establish the relationship between genotype and phenotype using whole-genome sequencing will require the integration of new mapping methods, the generation and analysis of multiple independent alleles, and functional assays. This will be especially challenging in natural populations, given extensive phenotypic and sequence diversity, as is already apparent from the genome sequences of James Watson and J. Craig Venter [14],[15]. If given their unlabeled genomes, we would not even be able to tell which one belonged to whom, much less provide a detailed accounting of which of the millions of sequence differences are responsible for which phenotypic traits.

For microbial organisms, the day is almost here when genome sequencing will be as routine as streaking out a strain. Within five years, resequencing whole genomes will be a part of the everyday world of biologists working on any organism. Some of the initial applications are obvious: already, high-throughput sequencing has been applied to improve measurements of global transcription factor binding, transcriptional profiles, and DNA methylation status [16]. What's more exciting is that radically increased sequencing capacity will likely lead to the development of entirely new and unforeseen methods for interrogating biology—much like the myriad applications that PCR has enabled. New approaches and new questions are certain to follow. Genomics, like molecular biology before it, will complete its transformation from a narrow subdiscipline—accessible to only a few—to a ubiquitous part of the biologist's toolkit.
